# Reply to Cosgrove: Catalytic activity in expansin-like proteins does not explain wall creep but raises a flag

**DOI:** 10.1073/pnas.2617956123

**Published:** 2026-07-17

**Authors:** Ignacio Delgado Santamaría, Vincent G. H. Eijsink, Anikó Várnai

**Affiliations:** ^a^ Faculty of Chemistry, Biotechnology and Food Science, Norwegian University of Life Sciences, Ås 1432, Norway

In a thoughtful letter (1), Cosgrove comments on our discovery that a single-domain fungal expansin-like protein (ELP) called *Gt*EXPN_133317 has catalytic activity on xylan (2). He points out that our findings do not prove that catalysis plays a role in wall creep caused by canonical two-domain expansins and rightfully adds that wall creep has never been demonstrated for single-domain ELPs, i.e., proteins that contain the DPBB domain but lack the CBM63 domain.

In our paper, we distinguish between canonical two-domain expansins and single-domain ELPs such as *Gt*EXPN_133317. We do not dispute the importance of the CBM63 domain for substrate binding and the activity that drives creep (i.e., the irreversible extension of growing plant cells under constant stress) in canonical expansins, as Cosgrove’s work has clearly demonstrated (3). We do not claim that catalysis *causes* creep; we claim that catalysis *exists* among proteins from the expansin superfamily. Cosgrove does not dispute this latter finding, and he is right when pointing out that there is no proof linking this catalytic activity to wall creep.

While we fully agree with Cosgrove that there currently is no evidence linking catalysis, i.e., the ability to cleave plant cell wall polysaccharides, to wall creep, we do not wish to exclude the possibility that such a link exists. These are intriguing proteins (4) acting on complex co-polymeric plant cell wall structures, the exact molecular nature of which is not known. The products of catalytic action, if any, may be hard to find because cleavage at only specific sites in the cell wall generates products that cannot be detected even with sensitive methods. With experience from working on *Gt*EXPN_133317 (2) and, previously, on LPMOs (5, 6), and being intrigued by the question of how canonical expansins do their job, we do not dismiss the possibility that catalytic action is involved in wall creep.

The extensible primary cell wall of growing plant cells that plant expansins work on is fundamentally different from the more rigid cell wall of differentiated plant cells that microbial expansins and ELPs mainly encounter (7, 8). As we discuss in our original paper (2), and as pointed out by Cosgrove (1), the low xylanolytic activity detected for *Gt*EXPN_133317 is not compatible with the rapid induction of creep seen for canonical expansins acting on primary cell walls. However, the low rate does seem compatible with the slower “weakening” effects on secondary cell walls observed for microbial expansins and ELPs (9, 10), including *Pca*LOOL12, a close phylogenetic relative of *Gt*EXPN_133317. Cell wall loosening by these microbial ELPs may not manifest in creep because they are acting on the cell walls of differentiated plant cells. These observations and considerations underpin that ELPs and plant expansins may act via distinct mechanisms, but do not as such exclude the possibility that catalysis plays a role in canonical expansins.

Biomechanical characterization of *Gt*EXPN_133317 and its catalytic mutants and assessment of catalytic activity in other members of the expansin superfamily should be key priorities for further research in this field.

## Declaration of Generative AI and AI-Assisted Technologies

During the preparation of this work, the authors used Claude (Anthropic) and Perplexity (Perplexity AI) for textual refinement and critically assessed any AI-generated content. The authors take full responsibility for all conceptual arguments, critical interpretations, and scientific conclusions presented in the paper.

## Data Availability

There are no data underlying this work.
